# Scalable Generation and Detection of on-Demand W States in Nanophotonic Circuits

**DOI:** 10.1021/acs.nanolett.3c01551

**Published:** 2023-05-24

**Authors:** Jun Gao, Leonardo Santos, Govind Krishna, Ze-Sheng Xu, Adrian Iovan, Stephan Steinhauer, Otfried Gühne, Philip J. Poole, Dan Dalacu, Val Zwiller, Ali W. Elshaari

**Affiliations:** † Department of Applied Physics, KTH Royal Institute of Technology, Albanova University Centre, Roslagstullsbacken 21, 106 91 Stockholm, Sweden; ‡ Naturwissenschaftlich-Technische Fakultät, Universität Siegen, Walter-Flex-Straße 3, D-57068 Siegen, Germany; § National Research Council of Canada, Ottawa, Ontario K1A 0R6, Canada

**Keywords:** Nanowire Quantum Dots, Single Photons, Multipartite Entanglement, W-State, Phase Retrieval, Gerchberg-Saxton Algorithm

## Abstract

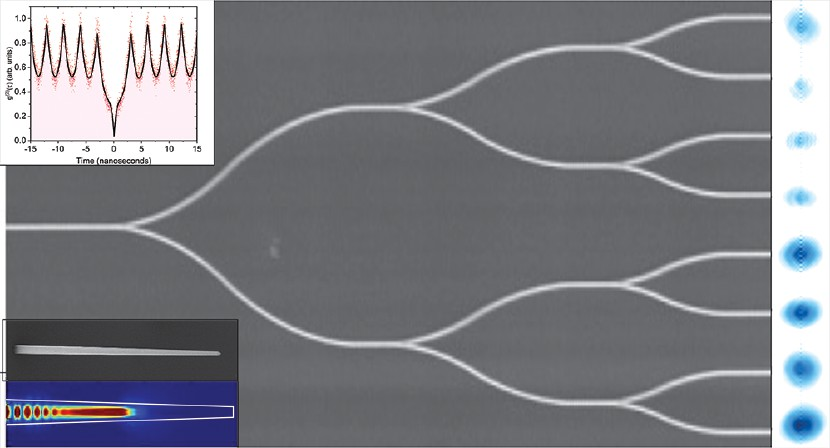

Quantum physics phenomena, entanglement and coherence, are crucial for quantum information protocols, but understanding these in systems with more than two parts is challenging due to increasing complexity. The W state, a multipartite entangled state, is notable for its robustness and benefits in quantum communication. Here, we generate eight-mode on-demand single-photon W states, using nanowire quantum dots and a silicon nitride photonic chip. We demonstrate a reliable and scalable technique for reconstructing the W state in photonic circuits using Fourier and real-space imaging, supported by the Gerchberg-Saxton phase retrieval algorithm. Additionally, we utilize an entanglement witness to distinguish between mixed and entangled states, thereby affirming the entangled nature of our generated state. The study provides a new imaging approach of assessing multipartite entanglement in W states, paving the way for further progress in image processing and Fourier-space analysis techniques for complex quantum systems.

Correlations form the basis for scientific inferences about the world. One of the most notable examples is that of causal inference where correlations between events are explained in terms of models that relate them from direct causation and/or shared common cause.^1^ This is a central paradigm in data analysis in science (e.g, cosmology, medical and social sciences), whose results have an impact from our own understanding of reality to decision making in public policies. In all of these cases, probabilities (and consequently, correlations) arise due to ignorance about all of the parameters behind the analyzed events. In contrast, entanglement is a particular type of correlation between space-like separated quantum systems for which there is no counterpart in the classical world. This fact is precisely stated by Bell’s theorem,^2^ which demonstrates the impossibility of reproducing correlations between measurement results performed on entangled quantum systems in terms of local-hidden-variable models, a result whose experimental verification and impact on quantum information science led to the 2022 Nobel Prize in Physics.

The characterization and detection of entanglement, as well as their impact in subsequent generation and experimental manipulation, is therefore of paramount importance. Such questions, however, are equally challenging, both theoretically and experimentally. These difficulties are particularly accentuated when we consider entanglement between more than two particles, here called multipartite entanglement. A fundamental problem lies in the exponential scaling of the dimension of the underlying Hilbert space, thus making an exhaustive classification difficult. In order to gain insight into multipartite entanglement phenomena, different concepts based on symmetries, graphical representations, matrix-product approximations, etc. have been used to select quantum states with particularly relevant properties within some context (see, e.g., refs (3−5).). In this work, we are interested in the so-called W states of *N* qubit systems. These states are characterized by a coherent superposition of all the qubits involved, with equal probability amplitudes. They gained prominence in the scientific literature in the context of multipartite entanglement classification.^6^ As it turns out, such states are intrinsically robust against particle loss and have been shown to be central as a resource in quantum information processing and multiparty quantum communication.^5,7−12^ Furthermore, W states are examples of the so-called Dicke states, which are quantum states that arise naturally in the study of the emission of light by a cloud of atoms via so-called super-radiance.^13^

In the past two decades, the precise control of quantum systems allowed the experimental generation of multipartite entangled states.^14^ Several schemes have been presented for preparing W states in a variety of physical platforms, including cavity quantum electrodynamics,^15,16^ quantum spin chains,^17^ nuclear magnetic resonance,^18−20^ atomic systems,^21−26^ and trapped ions.^27,28^ These schemes are frequently not scalable and/or require complex quantum state witnesses or quantum state tomography for their analysis. Single photons in optical platforms, in contrast, can be generated and manipulated with a high degree of purity, which makes them promising candidates for high-order W state generation. The generation of W states on such platforms, however, is yet challenging since it typically requires complex bulk-optical setups.^29,30^

In this work, we propose a scalable method for generating and detecting W states in nanophotonic circuits. We experimentally generate an eight-mode W state on an integrated nanophotonic circuit based on cascaded arrays of Y-branch splitters. The circuit is fabricated on a complementary-metal-oxide semiconductor (CMOS)-compatible silicon nitride platform. On-demand single photons generated from an InAsP nanowire quantum dot are fiber-coupled onto the photonic chip with the nanophotonic circuit. The output facet of the chip is imaged, generating real and Fourier space images. We then employ the Gerchberg-Saxton phase retrieval algorithm^31^ to reconstruct the quantum state probability amplitudes and relative phases from the experimentally obtained real and Fourier space images. The experimentally obtained Fourier-space image is then compared with numerical simulations for the ideal case scenario of uniform coherent superposition. We observe a great similarity between these images, with both presenting similar interference patterns. Such a pattern is not presented by incoherent statistical mixtures, which leads us to conclude that the final state is indeed the W state.

Compared with previous experiments,^32^ our approach stands out for the on-demand nature of the quantum state generation, large operational bandwidth offered by the Y-splitter-based architecture, and the better scalability and smaller circuit size offered by our state analysis protocol.

We prepare the W state with an on-demand single photon source, as shown in the experimental setup in Figure 1a. The on-demand single photon source consists of an InAsP quantum dot (QD) embedded in a wurtzite InP nanowire,^33−37^ and further details on the corresponding nanowire growth process can be found in the Supporting Information. The nanowire quantum devices were maintained at 4.2 K in an *attocube* closed-cycle cryogenic system. The single photon source was excited using a 780 nm pulsed laser beam with a repetition rate of 320 MHz and an excitation power of 100 nW. A linear polarizer, a set of quarter-wave plates, and a half-wave plate are used to purify the laser’s polarization. From among 100s of tested ultrabright single photon sources, we selected the optimum emitter in terms of emission wavelength, brightness, and emission line width. A long pass filter is used to reject laser light from single photons emitted by the nanowire quantum dot. A cascade of adjustable long-pass, short-pass, and band-pass filters, placed on a rotating stage, are then used to select a single transition from the QD’s S-shell. After coupling the single photons to an optical fiber, the photons can be either connected to a Hanbury Brown and Twiss effect (HBT)^38,39^ setup to measure the second order correlation function or coupled, using a tapered optical fiber, to the photonic chip for W state generation. The output of the photonic chip is imaged using a qCMOS single photon sensitive camera by Hamamatsu. Real and Fourier space intensity images of the chip output can be projected to the camera using a combination of a 100× objective and an optical lens. Figure 1b and c show a scanning electron microscope image of the W state device and magnified image of a single Y-splitter.

**Figure 1:**
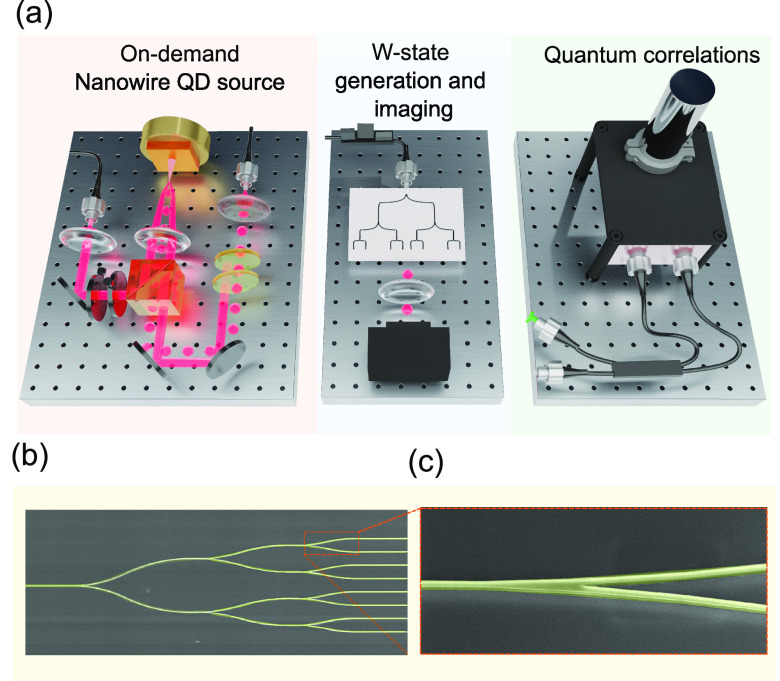
(a) Schematic of the experimental setup. The nanowire quantum dot (QD) is mounted in a closed-cycle cryogenic system operating at 4.2 K. The QD is excited using a tunable 320 MHz pulsed laser operating at a wavelength of 780 nm. The polarization of the laser is selected using a set of quarter-wave plates, a half-wave plate, and a linear polarizer. Single photons from the QD are filtered using a long pass filter for laser rejection, and a cascade of tunable long-pass, short pass, and band-pass filters mounted on a rotating stage to select a single transmission from the S-shell of the QD. The charged exciton line *X*^–^, shown in Figure 2, is then coupled to a single mode optical fiber. The single photons could be (1) coupled to a fiber-based 50:50 beam splitter with polarization controllers to a pair of superconducting nanowire single photon detectors for characterization of the second order correlation function or (2) coupled to the photonic-chip W state generator using a tapered optical fiber. The polarization of the single photons is set to the transverse electric (TE) field mode of the single mode photonic waveguide using a fiber-based polarization controller. The output of the chip is imaged using a 100× objective to a qCMOS Hamamatsu single-photon sensitive camera. The Fourier and real space images can be obtained by either adding or removing an additional optical lens before the camera. SEM images of the fabricated devices. (b and c) False-color SEM image of the representative eight-mode W state device and a single Y-splitter, respectively (the size of the imaged device is different from the actual one used for the experiment; the actual Y-splitter array has a total length of 1 mm and width of 26 μm at the output; an image of this aspect ratio would not be suitable to show the circuit details).

Figure 2a shows the emission spectrum of the nanowire quantum dot. The S-shell emission shows three types of particle complexes, a neutral exciton, biexcitons, and a trion, which were all verified using power and polarization series measurements. We used the brightest trion line at a wavelength of 881.7 nm, generating single photons with emission rates in the MHz range, measured using a superconducting single photon detector.^40−42^ To characterize the purity of the emitted single photons, we conducted zero delay second-order correlation measurement *g*^(2)^(0) using a fiber-based HBT setup equipped with two superconducting single photon detectors. The system efficiencies of the two detectors are 80% and 66%, with a timing jitter of 18 and 11 ps, respectively, and dark counts of less than 10 Hz. At zero delay, the measured value of *g*^(2)^(0) is 0.04, well below the high order emission level, allowing us to operate in the single photon-limit of the Hilbert space; the results are shown in Figure 2b. The nonzero value for the *g*^(2)^(0) is due to re-excitation of the quantum dot within the lifetime of the photon emission, and possible contributions from other states within the filtered emission range.

**Figure 2:**
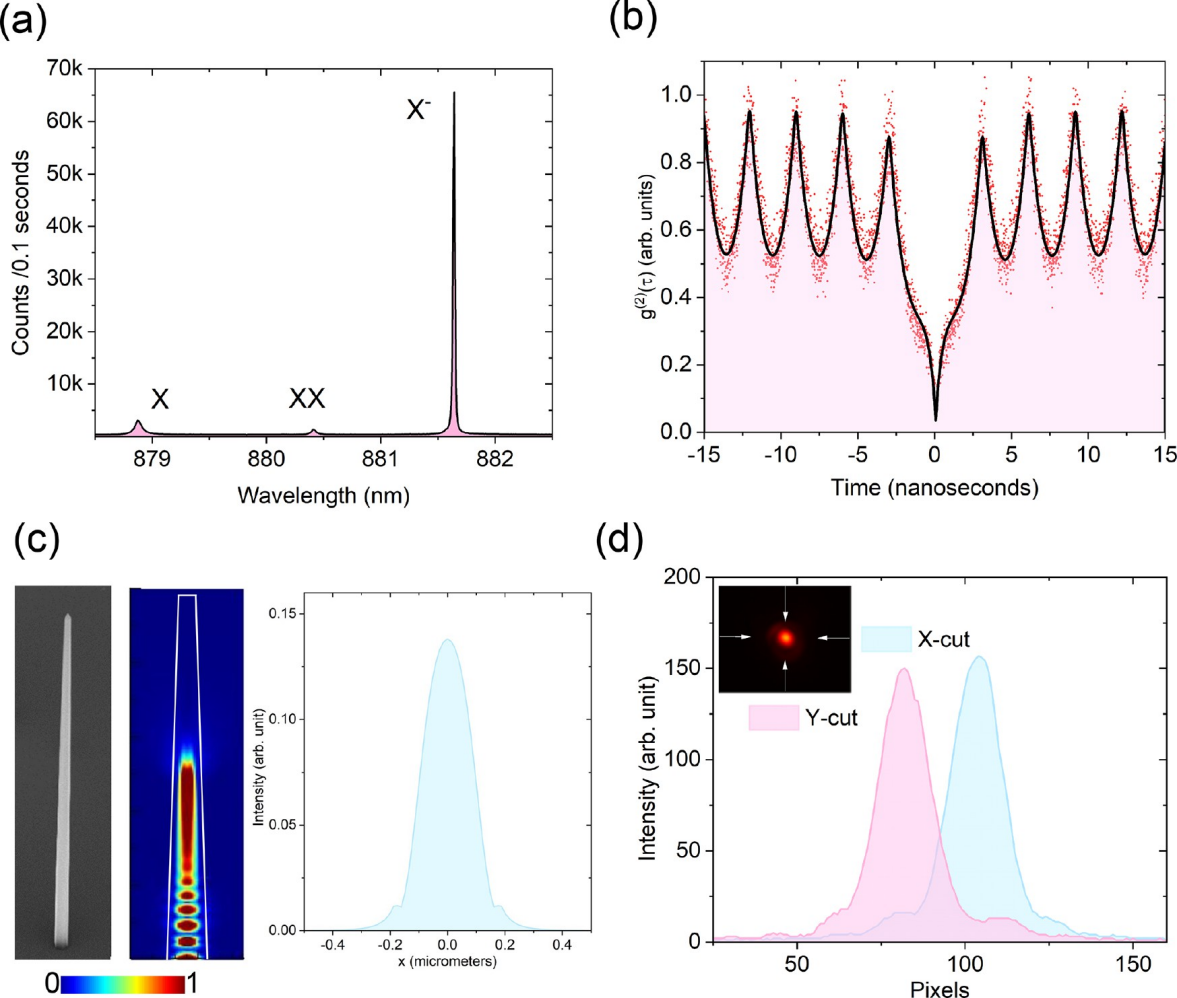
Nanowire-QD source. (a) Emission spectrum of the nanowire QD. At 100 nW laser excitation power, three emission lines are visible from the recombination of carriers in the S-shell of the QD. Using power and polarization series measurements, we identified the three emission lines to be the neutral exciton *X*, the biexciton *XX*, and the charged exciton (trion) *X*^–^. We observed that the charged exciton *X*^–^ is dominant under high energy laser excitation, consistent with previous measurements of similar nanowires. The nanowires exhibit excellent emission properties of small-line width and high brightness of several MHz detected on the superconducting nanowire single photon detectors. (b) Single photon purity. The second order correlation function *g*^(2)^(0) value at zero delay was measured to be 0.04. The black line shows fit to the experimental data points in red. The data are fitted with a series of correlation pulses at a repetition rate of 320 MHz weighted with an antibunching term at zero delay that describes the sub-Poissonoan statistics. (c) Numerical simulation of nanowire QD mode profile. 3D finite difference time domain simulations are performed to calculate the mode profile of the traveling waves in the nanowire. The source is a dipole oriented perpendicular to the growth direction, which is located 1.5 μm from the base of the nanowire. (d) Measured emission-profile of the nanowire QD. To facilitate the coupling to optical fibers with high efficiency, the emission profile of the single photons emitted from the nanowire was experimentally measured, following the numerical simulations. There is an excellent agreement with the numerical simulations, yielding a Gaussian-like profile thanks to the waveguiding effect provided by the core–shell structure of the nanowire QD.

To determine the mode profile of the single photons emitted from the nanowire, 3D finite difference time domain simulations were performed; the results are shown in Figure 2c. The QD is simulated as a dipole located 1.5 μm out from the base of the nanowire; the dipole orientation is perpendicular to the growth direction. The waveguiding, provided by the core–shell design and the tapering of the nanowire, forms a circularly symmetric mode profile that enhances coupling to single mode fibers. To verify the beam profile experimentally and enhance the coupling efficiency of the single photons to the optical fiber, the emission profile of the single photons was measured as shown in Figure 2d. The results show excellent agreement with the numerical simulations, with a Gaussian-like emission. The mode profile of the QD emission was matched to a 780HP single mode fiber using a Schäfter+Kirchhoff fiber coupler with an adjustable aspherical lens to achieve a high coupling efficiency.

The fiber-coupled single photons are then injected into the W state photonic chip using a tapered optical fiber with a working distance of 13 μm and spot size of 3 μm to maximize coupling to the transverse electric (TE) field mode of the photonic waveguides. The waveguides are made of silicon nitride deposited via the low pressure chemical vapor deposition (LPCVD) technique and then lithographically patterned to a width of 500 nm and a height of 250 nm, ensuring single mode operation at the nanowire quantum dot emission wavelength. The waveguides are coated with PMMA to ensure symmetric mode confinement. More details about the photonic chip fabrication can be found in the Supporting Information. The optical W state based on channel waveguides is characterized by a coherent distribution of a single photon over *N* waveguides. The state is defined by
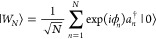
1where ϕ_*n*_ is the arbitrary phase and *a*_*n*_^†^ is the Bosonic creation operator at each channel. In our experiment, the single photon W state generation occurs through coherent evolution of single photons through cascaded arrays of three sets of Y-branch 50–50 power splitters. In our circuit, every Y-branching was made to be precisely transversely symmetrical, providing an identical path length from input to output, regardless of the path. The emission lifetime of our single photon source is on the order 1 ns, which is much longer than the path length corresponding to the physical dimensions of the chip. This ensures the presence of only a single photon in the chip at a time. In comparison to previously demonstrated methods employing directional couplers^32,43^ and evanescent coupling in waveguide arrays,^44,45^ the Y-splitters-based protocol is easier to design and scalable with a larger operating bandwidth, limited by the single mode cutoff of the photonic waveguide.

The single photon state, *a*_1_^†^|0⟩ (where *a*_1_^†^ is the creation operator of the input waveguide), launched into the input waveguide is initially localized. Its state after evolution through the first y-splitter can be expressed as a two-order W state:^46^
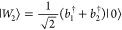
2where *b*_1_^†^ and *b*_2_^†^ are the creation operators at the outputs of the first Y branch.

Similarly, the state after the second set of two Y-splitters reads
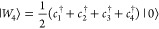
3and, finally, the final output state after the third set of four Y-splitters is the eight-order W state:
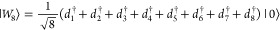
4Here, *c*^†^ and *d*^†^ are the creation operators for the second and third sets of Y-branch outputs, respectively. Therefore, as ideally a single photon is sent to the circuit, the final state produced will be an optical eight-order W state given by the above equation with equal relative phases [cf. eq 1].

After coupling the single photons to the chip input, the output facet of the chip was imaged using a qCMOS Hamamatsu camera. The Fourier and real space images can be obtained by either adding or removing an additional optical lens before the camera. In the experiment, we use the Gerchberg-Saxton algorithm, which was devised by crystallographers Ralph Gerchberg and Owen Saxton to deduce the phase distribution of electron beams in a transverse plane from the intensity distributions in two planes.^38,39^ A process flow diagram of the algorithm is shown in Figure 3. The output phase distribution of our circuit can be reconstructed using this iterative phase retrieval process. The algorithm takes the 2D matrix corresponding to the real space amplitudes *u*_0_ as the input and each point in the matrix is assigned an arbitrary phase value ψ. A Fourier transform operation on this matrix (with elements *u*_0_ exp(*iψ*)) gives a Fourier space matrix *U* exp(*i*Ψ) which can in turn give a real space matrix with the application of inverse Fourier transform on it. Several iterations of this scheme are performed, and each iteration yields a matrix with a set of either real space or Fourier space amplitudes and phases. After each transform operation, the amplitudes in the output matrix (*u*, *U*) are replaced by the amplitudes from the experimentally obtained real and Fourier images (*u*_0_, *U*_0_). These serve as the constraints in the algorithm. The phase values (ψ in real space and Ψ in Fourier space) are left unchanged and can evolve freely. The iterations continue until we get a convergence yielding real and Fourier space matrix amplitudes (*u*, *U*) very close to the experimentally observed ones (*u*_0_, *U*_0_). The phase that evolved freely now converges to certain values, which is equal to the actual relative phases of the real space image.

**Figure 3:**
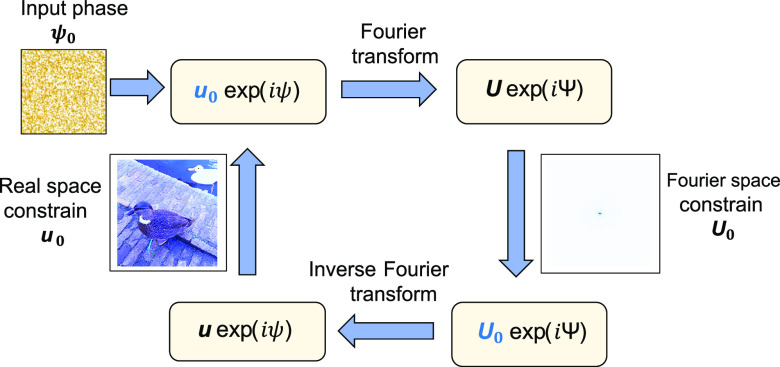
Work-flow diagram of the Gerchberg-Saxton algorithm. After the single photons are coupled to the photonic chip, intensity measurements of the real and Fourier space output-waveguide profiles are performed. The algorithms starts with the real space image initialized with a random phase distribution, serving as the basis for the optimization procedure. Then, while adhering to constrains imposed by the experimentally measured intensity distributions of the real and Fourier-space images, the Fourier and inverse Fourier transforms are carried out iteratively.

Figure 4a shows the real space amplitude (square-root of intensity) image of the output facet obtained using an exposure time of 10 min. Figure 4b shows the Fourier space image obtained using the same experimental setup. There are eight modes in the real space image corresponding to the eight-waveguides from the cascaded Y-splitters, and single photons are coherently distributed among them. This fact is highlighted in the Fourier space image by a distinct diffraction pattern produced by the individual photons (*g*^(2)^(0) = 0.04). The interference is the consequence of coherent single-photon superposition across the photonic chip without any knowledge of the which-path information. The real and Fourier space images in the experiment each had 4 million pixels; the Gerchberg-Saxton phase retrieval algorithm was run for 5000 iterations before convergence. The reconstructed amplitude and phase distributions of the W state from the experiment are shown in Figure 4c and d, respectively. The algorithm was able to successfully reconstruct the eight-mode real space image by directly taking the inverse Fourier transform of the reconstructed Fourier-space image. The degree of similarity of the reconstructed real space image with the experimentally obtained real space image is a measure of the accuracy of the derived phase values. Figure 4e and f show extracted amplitudes and phases of the experimentally measured on-demand W state generated by our photonic chip. We could observe good uniformity in the output probability amplitudes with a standard deviation of 0.085 around the mean value of 0.343 and a standard deviation of 0.086 around the ideal value of 0.354. The obtained phase values are also close to the ideal value of 0. The finite deviation from the ideal values in both cases is mainly due to the slight imperfections in the fabricated nanostructures and background noise scattered in the cladding during image acquisition.

**Figure 4:**
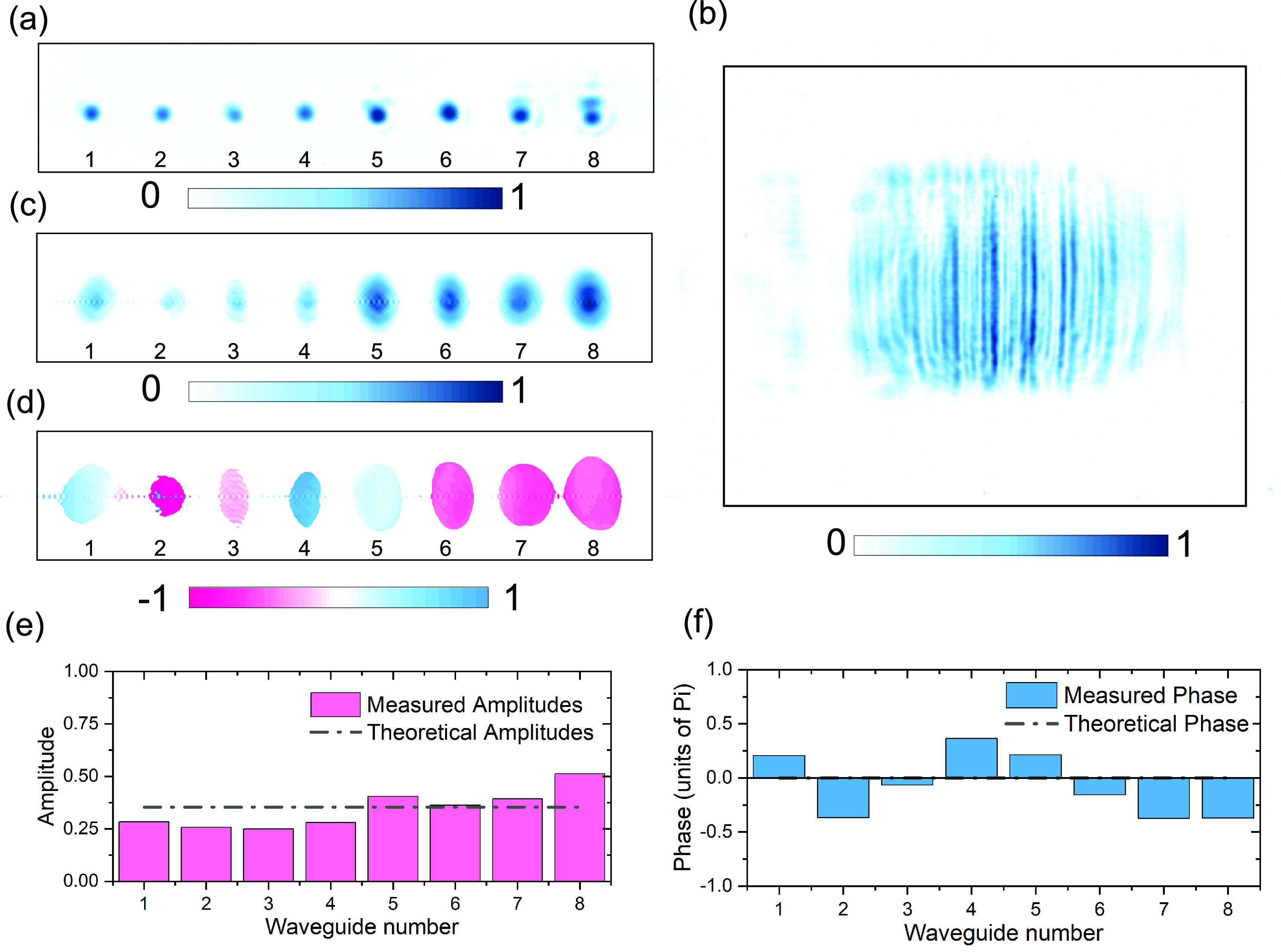
Quantum state reconstruction. (a and b) Square-root of the real and Fourier space images. The real space image consists of eight modes, with single photons coherently distributed between them. The Fourier space image emphasizes this fact through a clear diffraction pattern created by the single photons. The interference pattern results from the coherent propagation of single photons through the chip, with no which-path information available. The images were recorded for an integration time of 10 min. (c and d) Reconstructed amplitude and phase profiles of the real space image. The starting real and Fourier space images each contain 4 million pixels; the algorithm converged after 5000 interactions. There is a good agreement between the reconstructed and measured real space images; deviations in the reconstructed image are attributed to noise during data collection, which can be further reduced with enhanced coupling to the photonic chip. The phase in d is measured in units of π. (e and f) Extracted amplitudes and phases of the W state. The theoretical values of the amplitude (1/√8) and phase (0) are highlighted by a black-dotted line.

Traditionally, W states have been identified through state tomography and entanglement witnesses. Proper implementation of such techniques allows rigorous verification of the presence of multipartite entanglement or even reconstructing the obtained state. However, the complexity of implementing these techniques prohibits their application for quantum states involving a high number of qubits. So, it is desirable to find a scalable approach for higher-order W state verification. Here, we propose a simple method based on image comparison techniques combined with reasonable assumptions about the experimental setup.

We start with some optical eight-order *W* state [eq 5]: 8^–1/2^∑_*n*=1_^8^ exp(*iϕ*_*n*_) *a*_*n*_^†^ |0⟩. The output image in the real space is composed of eight spots, each one corresponding to a term exp(*iϕ*_*n*_) *a*_*n*_^†^ |0⟩. The Fourier space image, in turn, is obtained via
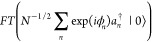
5where FT(|ψ⟩) stands for the Fourier transform of the image generated by |ψ⟩. In the case of an ideal W state, 8^–1/2^∑_*n*=1_^8^ *a*_*n*_^†^ |0⟩, the real space image corresponds to eight identical Gaussian spots, and the Fourier space image resembles that of the *n* slits experiment with a characteristic interference pattern arising from the coherent superposition between different distinguishable states. In contrast to this, if the state undergoes complete decoherence, its real space image remains the same, but the interference pattern in the Fourier space image disappears since its Fourier transform now reads
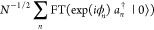
6

In Figure 5, we compare the ideal W state image, its fully decohered version (mixed state), and the experimentally obtained image. Visually, we can clearly see that the result obtained experimentally resembles the simulation for the ideal W state, being in strong contrast with the mixed state. This is indeed confirmed since the images are more than 90% similar when compared via the structural similarity index measure (SSIM).^47^ Furthermore, by computing the correlation between these images, we can estimate the overlap between the ideal W state and the experimentally produced state, from which we get 83.1%. We now employ an entanglement witness of the form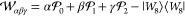 (where  is the projection onto the subspace with *i* excitations) under different assumptions. By assuming that the produced state is a convex mixture of the ideal W state and its fully decohered version, i.e., , we get *p* > 80%. Employing the methods described in ref (5) and briefly reviewed in the Supporting Information, we numerically found  witnessing the entanglement of the generated state. Moreover, the value of the second order correlation function *g*^(2)^(0) at zero delay gives an upper bound on the probability of having more than one photon on the chip. It was experimentally measured (Figure 2b) to be 0.04. Considering multiple photon generation as another possible source of noise in the generated W state, the final state would have the form  if we neglect contributions from subspaces with more than two excitations. These states, with the value of *q* upper bound by 0.04, can also have their entanglement witnessed by .

**Figure 5:**
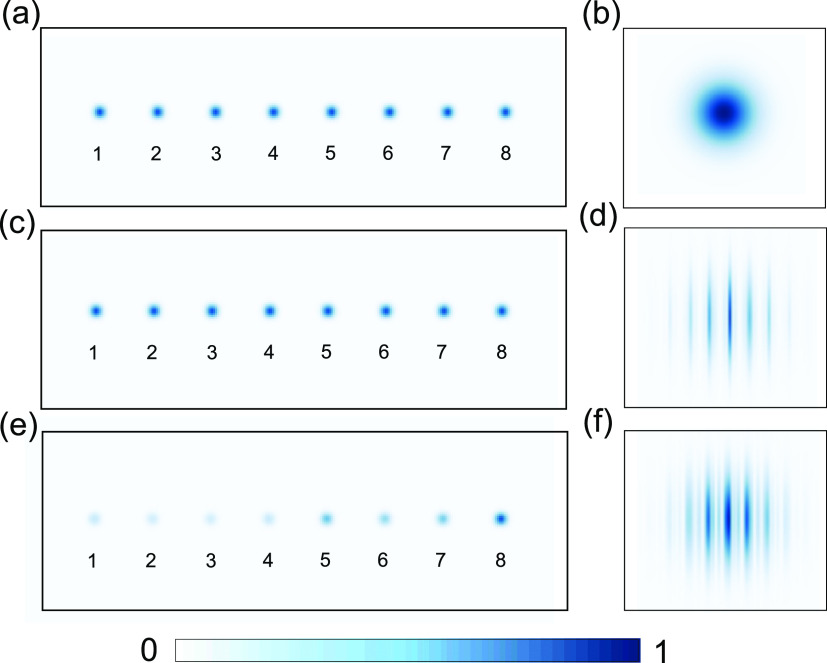
Verification of the multipartite coherent superposition. (a and b) Real and Fourier space images of a mixed state. Eight-mode single photon mixed state is numerically generated, then its Fourier transform was computed. The interference pattern in the Fourier transform vanishes, yielding a Fourier transform profile corresponding to a single mode of the input. (c and d) Real and Fourier space images of the pure W state. The eight-mode single photon W state is numerically generated with equal amplitudes of 1/√8 and a relative phase of 0 between different modes. The Fourier transform shows a clear interference as opposed to the mixed state case. (e and f) Real and Fourier space images experimentally measured the W state. Using the experimentally measured phases, we numerically constructed the W state in the experiment and its Fourier transform. We clearly see an interference pattern in the Fourier transform, in close agreement with the measurements in Figure 3. This is in clear contrast to the theoretical results of a mixed state shown in Figure 4a.

We have demonstrated a scalable on-demand scheme for high-order on-chip single-photon W state generation. The on-demand nature of our protocol, using nanowire QDs and a silicon nitride hybrid system, facilitates the scope of its integrability into other hybrid quantum systems,^48,49^ with potential bit rates in the GHz range, limited only by the lifetime of the quantum emitter. Our circuit based on Y-splitters uses no resonant or interference effects, thus delivering large operating bandwidth. The ease of fabrication and the Fourier-space imaging-based verification of superposition and coherence make our approach scalable to higher-order W states. Through experimental measurements and theoretical modeling, we showed strong evidence to verify that our output state is a multipartite coherent superposition, as opposed to a mixed state in which interference between different channels vanishes. Our findings pave the way for future developments in image processing methods and Fourier space analysis for characterizing multipartite entangled quantum systems. Multipartite entanglement provides a great deal of room for phenomena that are not available in systems with just two subsystems, which makes it an active field of research, from both fundamental science and applications points of view.^50−52^ Our results introduce a quantifiable visual approach to experimentally validate multipartite entanglement, which can be of paramount importance for the experimental advancement of multiparticle quantum-information processing protocols.

## Supporting Information Available

Quantum entanglement of W states, photonic circuit fabrication, nanowire QD growth, Fourier-space images of W states, entanglement witnesses (PDF)
