# Supplementary material for: Scalable Generation and Detection of on-Demand W States in Nanophotonic Circuits
Source: Nano Lett. 2023 May 24;23(11):5350–7. doi: 10.1021/acs.nanolett.3c01551 (PMC10273479; doi:10.1021/acs.nanolett.3c01551)
Supplement: Supplementary file 1 — nl3c01551_si_001.pdf [file nl3c01551_si_001.pdf]

Supporting Information: Scalable generation and detection of on-demand W states in nanophotonic circuits.

Jun Gao[1,†,‡,*], Leonardo Santos[2,†], Govind Krishna[1,†,‡,*],
Ze-Sheng Xu[1], Adrian Iovan[1], Stephan Steinhauer[1], Otfried Gühne[2], Philip J. Poole[3],
Dan Dalacu[3], Val Zwiller[1] & Ali W. Elshaari[1,‡,*],

[1]Department of Applied Physics, KTH Royal Institute of Technology, Albanova University Centre, Roslagstullsbacken 21, 106 91 Stockholm, Sweden
[2]Naturwissenschaftlich-Technische Fakultät, Universität Siegen, Walter-Flex-Straße 3, D-57068 Siegen, Germany
[3]National Research Council of Canada, Ottawa, Ontario, Canada, K1A 0R6
[†]Equal contribution
[‡]E-mail: junga@kth.se, govindk@kth.se, elshaari@kth.se
[*]Corresponding author

Quantum entanglement of W-states

Here, we briefly review quantum entanglement theory with a focus on W states. We restrict ourselves to two-level quantum systems (qubits). As usual, we fix a "computational basis" defined by two orthogonal states: $|0\rangle$ and $|1\rangle$. To start with, we consider the simplest bipartite quantum system presenting entanglement, a pair of qubits A and B. A given pure state $|\psi\rangle_{\mathsf{AB}}$ is entangled if it cannot be written as a product, i.e., $|\psi\rangle_{\mathsf{AB}} \neq |\phi\rangle_{\mathsf{A}} \otimes |\tau\rangle_{\mathsf{B}}$ for all local states $|\phi\rangle_{\mathsf{A}}$ and $|\tau\rangle_{\mathsf{B}}$.

For two quantum states, one can ask more generally, whether one state can be transformed into the other via local operations and classical communication (LOCC) [1]. For pure bipartite states this can be solved [2], but in the general case this is a hard and open question [3].

A slight, but significant generalization of LOCC is that of stochastic local operations and classical communication (SLOCC). These are LOCC transformation on a single copy of a state, but without imposing that the target state has to be achieved with certainty. In that case, two states are equivalent if each one of them can be converted into the other with a non-zero probability and vice versa. For two qubits there is only one equivalence class represented by the

Bell state:

$$|\Phi^+\rangle = \frac{1}{\sqrt{2}}(|00\rangle + |11\rangle). \tag{S1}$$

Any entangled state can be obtained via LOCC from the Bell state, and conversely, any entangled state can be transformed into the Bell state with nonzero probability via SLOCC. This is one of the many facts that justify the designation of "maximally entangled" for the Bell state.

When considering a system of three or more qubits, the situation becomes much more complex. For three qubits A, B and C, for example, a pure state $|\psi\rangle_{\mathrm{ABC}}$ may be written as a product in two different ways: total separability, when the state is written as a product of three local states, $|\psi\rangle_{\mathrm{ABC}} = |\alpha\rangle_{\mathrm{A}} \otimes |\beta\rangle_{\mathrm{B}} \otimes |\gamma\rangle_{\mathrm{C}}$; and biseparability if $|\psi\rangle_{\mathrm{ABC}} = |\phi\rangle_{\mathrm{A}} \otimes |\tau\rangle_{\mathrm{BC}}$, $|\psi\rangle_{\mathrm{ABC}} = |\phi\rangle_{\mathrm{AB}} \otimes |\tau\rangle_{\mathrm{C}}$ or $|\psi\rangle_{\mathrm{ABC}} = |\phi\rangle_{\mathrm{B}} \otimes |\tau\rangle_{\mathrm{CA}}$. If $|\psi\rangle_{\mathrm{ABC}}$ is neither fully separable nor biseparable then it is genuine multipartite entangled [4]. Furthermore, pure genuine multipartite entangled states can be entangled in two inequivalent ways [5], i.e., there exist two classes of states which cannot be transformed into another by SLOCC, in contrast to two qubits. The representatives of these entanglement classes are the Greenberger-Horne-Zeilinger (GHZ) state,

$$|\mathrm{GHZ}\rangle = \frac{1}{\sqrt{2}}(|000\rangle + |111\rangle), \tag{S2}$$

and the W state

$$|\mathrm{W}\rangle = \frac{1}{\sqrt{3}}(|100\rangle + |010\rangle + |001\rangle). \tag{S3}$$

The GHZ and W states are of central importance in quantum information science [4, 6]. Both can lead to violations of Bell inequalities [7, 8], with the GHZ state violating the famous Mermin inequality [9] maximally and leading to the GHZ argument [10]. Contrary to that, the entanglement in the W state is robust against particle loss and the state is maximally entangled according to the geometric measure of entanglement [11, 12].

Both states can be generalized to systems with many qubits. However, for systems with more than three qubits, there are infinitely many equivalence classes via SLOCC, which makes

characterization much more complex. The generalization of Eq. (S8) for N qubits reads

$$|\mathsf{W}_N\rangle = \frac{1}{\sqrt{N}}(|100\ldots0\rangle + |010\ldots0\rangle + \cdots + |000\ldots1\rangle). \tag{S4}$$

This state presents a variety of properties that make it unique in the set of pure states of many qubits system. The first of these properties is that, although it generally does not lead to the maximum violation of the better-known Bell inequalities in contrast to the GHZ state, the W state is much more robust against particle loss [4], making W state a good candidate to encode quantum information. In fact, the marginal states of GHZ (S6) are separable while the W state (S8) is the state with maximum possible bipartite entanglement in the reduced two-qubits state. Last but not least, W states are high-dimensional quantum states that exhibit a high degree of entanglement and whose experimental generation can be implemented robustly and much less demanding than other quantum states (e.g., GHZ states). So, on-demand generation of W states is a valuable tool for quantum technologies since such states are highly entangled and quite robust against harmful effects of the surrounding environment.

Photonic circuit fabrication

To design the waveguide, ellipsometry measurements to characterise the height of the silicon nitride and its refractive index were performed. The simulated mode profiles based on the experimental measurements are shown in Fig. S1(a), for the transverse electric TE (a) and transverse magnetic TM (b) modes. The TM mode is weakly localized, with an effective index of 1.588, compared to the TE mode which has an effective index of 1.643. The simulations are performed at the emission wavelength of the trion line of the QD.

The substrate consists of a 500 μm thick silicon wafer capped with 3.3 μm of thermal oxide, and 250 nm LPCVD silicon nitride prepared by Rogue Valley Microdevices. An adhesion promoter, AR 300-80 by ALLRESIST, is spin-coated on the substrate at 3000 RPM. The substrate is then soft-baked for 90 seconds at 90 °C. Negative resist m-aN 2403 by Microresist

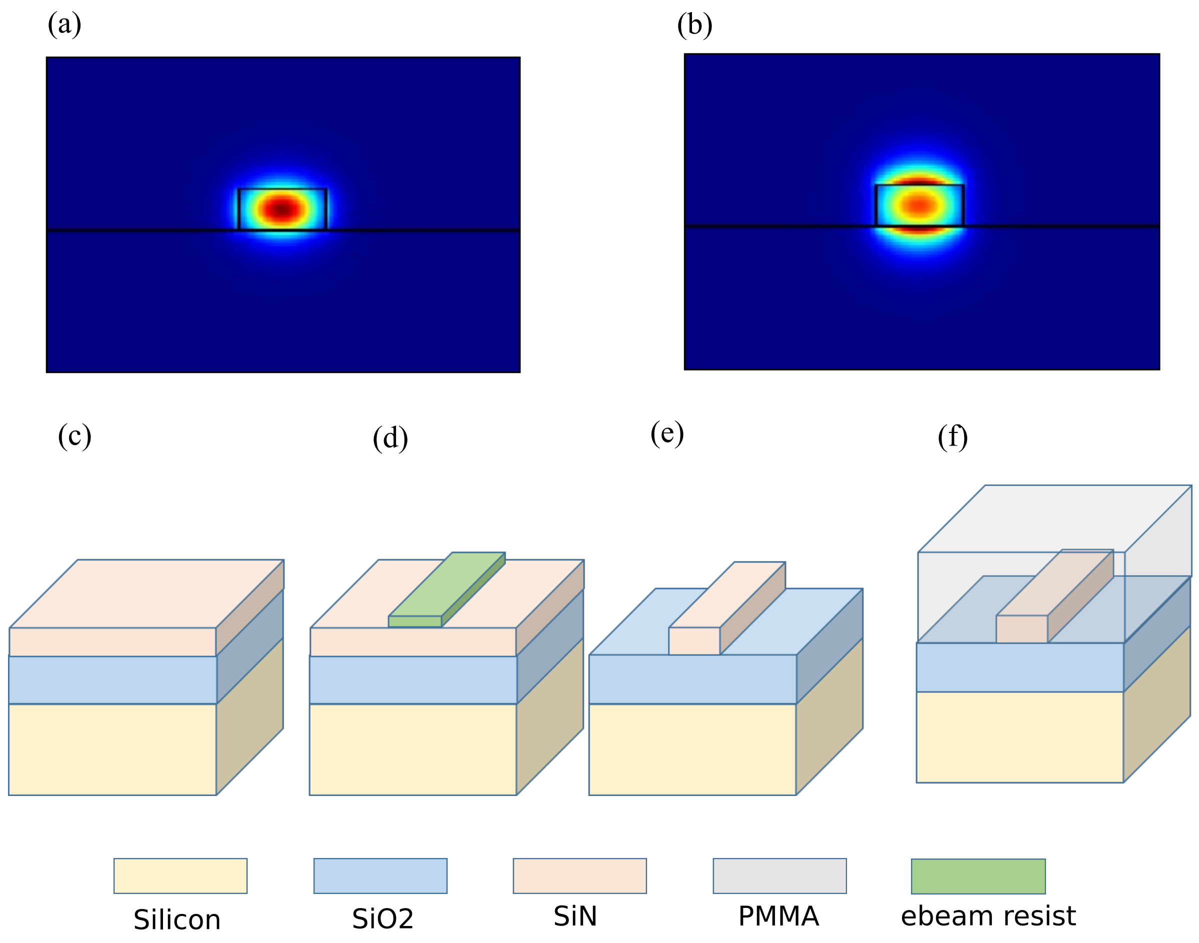

Figure S1: **Mode profiles.** (a) and (b) show Horizontal and vertical electric field components for the fundamental TE and TM modes, respectively. **W state chip fabrication process**. (c) Substrate consisting of a silicon wafer, 3.3 μm of silicon oxide and 250 nm of silicon nitride. (d) electron beam lithography to define the pattern. (e) Reactive ion etching to form the circuit in the silion nitride layer. (f) top cladding of the chip with PMMA for symmetric mode confinement.

technology is spin-coated at 3000 RPM and baked at 90 $^{\circ}$C for 1 minute, yielding an approximate resist thickness of 300 nm. The electron beam lithography dose assignment in the CAD is proximity-corrected using commercial software package BEAMER. The cad is exposed using 50 keV electron-beam lithography Voyager system developed by Raith nanofabrication. The waveguide width was designed to be 600 nm. After exposure, the chip is developed in ma-D 525, an aqueous-alkaline based developer, supplied by Microresist technology, then the chip is rinsed in DI water. The waveguides are etched in PlasmaPro 100 Cobra ICP etching System using SF6 based chemistry. After etching, 950K A8 PMMA resist was spin-coated at 1000 RPM and baked at 150 $^{\circ}$C for 5 minutes. The refractive index of PMMA at 885 nm, the emission wavelength range of the S-shell transitions in the QD, is closely matched to the bottom oxide cladding. This provides symmetric mode confinement of the single photons in the silicon nitride waveguide. Finally, the chip is cleaved through a crystallographic direction of the silicon wafer, providing a smooth chip-facet for coupling light into the waveguides using a tapered optical fiber. The fabrication steps are depicted in Fig. S1(d) to (f).

Nanowire QD growth

Chemical beam epitaxy using Trimethylindium (TMI), phosphine (PH_3) and arsine (AsH_3) as sources of In, P and As, respectively, was used to grow the wurtzite nanowires for this study. We us a selective-area vapour-liquid-solid growth technique described in detail in Refs. [13, 14, 15]. On a (111)B InP substrate we deposit a 20 nm thick SiO_2 mask. Using electron-beam lithography, HF wet-etching and metal lift-off we produce patterned substrates consisting of gold droplets in the centres of holes in the SiO_2 mask. On this substrate we first grow InP nanowire cores which have a diameter of 20 nm, set by the droplet size. In these cores we incorporate InAsP quantum dots ~ 5 nm thick and having the same diameter as the core. We then clad the core with an InP shell to produce a photonic nanowire having a base diameter of 250 nm

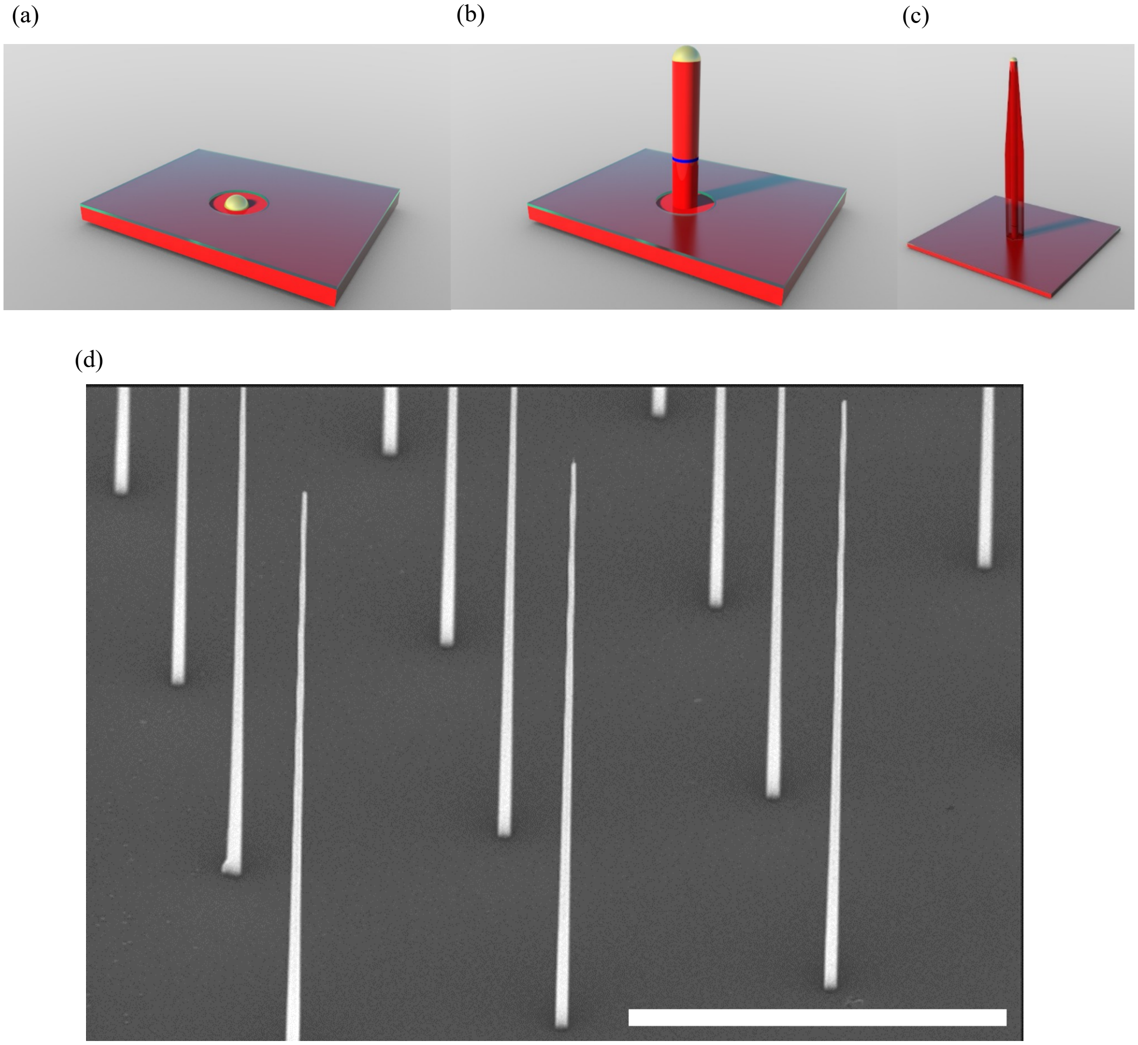

Figure S2: **Growth process of the nanowire quantum dot source.** (a) The process starts with a deterministically placed gold droplet to mediate the nanowire growth. (b) and (c) show the growth processes for the core, QD and cladding by controlling the chemistry and temperature, as highlighted in the text. **SEM of nanowires array**. (d) The scale bar has a length of 10 μm. The controlled spacing between the nanowires allows for addressing a single nanowire-quantum dot using the laser.

which tapers to 100 nm over the $\sim 15\,\mu$m length of the nanowire. The cladding is produced by adjusting the growth conditions from that used to grow the core, in particular, increasing the growth temperature from 435° to 450° and increasing the V/III ratio. The growth process is shown in Fig. S2(a)-(c). A scanning electron microscope image of an array of deterministically fabricated nanowire quantum dots is shown in Fig. S2(d).

Fourier-space images of W states

The quantum interference between different channels in our system, which is revealed by the Fourier-space image, resembles that of n-slit experiment. To reveal this similarity, we constructed different quantum W states and computed their Fourier transform as shown in Fig. S3. We selected W states of the following orders:

$$\begin{aligned}
&|\mathrm{W}\rangle = \frac{1}{\sqrt{1}}(|1\rangle)\\
&|\mathrm{W}\rangle = \frac{1}{\sqrt{2}}(|10\rangle + |01\rangle)\\
&|\mathrm{W}\rangle = \frac{1}{\sqrt{4}}(|1000\rangle + |0100\rangle + |0010\rangle + |0001\rangle)\\
&|\mathrm{W}\rangle = \frac{1}{\sqrt{6}}(|100000\rangle + |010000\rangle + |001000\rangle + |000100\rangle + |000010\rangle + |000001\rangle)\\
&|\mathrm{W}\rangle = \frac{1}{\sqrt{8}}(|10000000\rangle + |01000000\rangle + |00100000\rangle + |00010000\rangle + |00001000\rangle + |00000100\rangle\\
&+ |00000010\rangle + |00000001\rangle).
\end{aligned} \tag{S5}$$

In the trivial case of a single Gaussian beam input $g(x, y)$, W state of the 1st order, the far-field diffraction is the Fourier transform (FT) of the input mode. The Fourier transform is a scaled Gaussian function, but in the spatial frequency space (f_x, f_y). The results are shown in Fig. S3(a) and (b) and described by

$$\text{Interference pattern} = FT[g(x, y))] = G(f_x, f_y). \tag{S6}$$

The situation becomes more interesting when more quantum channels are involved. For example, in the 2nd order W state, we can use the the translation property of the Fourier transform. If we assume that the two input modes are located at distances $\pm d$ from zero in the $x-$direction, the interference pattern can be written as

$$\text{Interference pattern} = FT[g(x-d,y)+g(x+d,y))] = G(f_x,f_y)[e^{j2\pi f_x d}+e^{-j2\pi f_x d}] \tag{S7}$$

The intensity of the interference pattern is simply a Gaussian function modulated by a squared cosine function. The input mode profile and the diffraction pattern intensity profile are shown in Fig. S3(c) and (d) and given by

$$\text{Intensity profile} \sim G(f_x,f_y)\cdot \cos^2(2\pi f_x d). \tag{S8}$$

As the number of modes is increased, we can write the diffraction pattern of the W state as

$$\text{Intensity profile} \sim G(f_x,f_y)\cdot \frac{\sin^2(N\pi f_x d)}{\sin^2(\pi f_x d).} \tag{S9}$$

Here, N is the number of interfering modes in the W state in Eq. S5. The position of the bright regions in the Fourier transform is preserved, following the same physics as in multi-slit diffraction. The results for the cases of 2, 4, 6, and 8 modes are shown in Fig. S3(c) to (k). This is in a stark contrast to the mixed-state case, where the interference between different modes in the W state is lost, resulting in an incoherent mix of all the modes, with vanishing diffraction pattern.

In our setup the Fourier transform can be computed by inserting a lens to project the W state output of the chip to the back-focal plane of the lens. The image in the back-focal plane of a positive lens, having a focal length f and light of wavelength λ, is given by

$$G\left(x\prime,y\prime\right) = \exp\left\{j\pi\frac{x\prime^2+y\prime^2}{\lambda f}\left(1-\frac{z}{f}\right)\right\}\iint g(x,y)\exp\left\{-j2\pi\frac{xx\prime+yy\prime}{\lambda f}\right\}\mathrm{d}x\,\mathrm{d}y, \tag{S10}$$

where $x\prime$ $y\prime$ are the transverse spatial coordinates after the lens at a distance z. The output image is simply the 2-dimensional Fourier transform of the input, as shown by the integral over

the input state in the second term. The integration is taken over the pupil function of the lens. The first term describes the spherical wave-front free-space propagation.

When the camera is placed exactly one focal distance behind the lens, the Fourier transform computed by the lens is exactly

$$G\left(x_f, y_f\right) = \iint g(x, y) \exp\left\{-j2\pi \frac{x x_f + y y_f}{\lambda f}\right\} \mathrm{d}x\, \mathrm{d}y, \tag{S11}$$

where the spatial frequencies in the f_x and f_y direction are related to the spatial coordinates at the focal point x_f, y_f by $f_x = x_f/\lambda f$ and $f_y = y_f/\lambda f$. Moreover, the translation property of the Fourier transform can be understood in our optical setup as shown in Fig. S4. Different modes of the W state are focused to the back-focal plane, with each having a different path-length corresponding to unique phase factor in the Fourier transform. This coherent locked phase between the modes enables the diffraction pattern we measure in the experiment between different single-photon channels.

Entanglement witnesses

An entanglement witness is a self-adjoint operator $\mathcal{W}$ satisfying tr$(\mathcal{W}\rho) \geq 0$ for all density operator representing a separable (i.e., non-entangled) quantum state. Thus, tr$(\mathcal{W}\rho)$ being negative is a sufficient criterion to conclude that a given state ρ is entangled. The construction of an adequate entanglement witness depends on some prior knowledge about the devices that produce such a quantum state. Here, in particular, we ideally produce the 8 order W state, $|W_8\rangle$. In that case, a good ansatz for entanglement witness is [16, 4]

$$W_{\alpha\beta\gamma} = \alpha\mathcal{P}_0 + \beta\mathcal{P}_1 + \gamma\mathcal{P}_2 - |W_8\rangle\langle W_8|. \tag{S12}$$

Here $\mathcal{P}_i$ are projectors onto the subspaces with exactly i excitations. We need to guarantee that $\mathcal{W}_{\alpha\beta\gamma}$ is actually an entangled witness. From the symmetry of the W state, it suffices to prove non-negativity for states $|a\rangle \otimes |b\rangle$, where $|a\rangle = a_0|00...00\rangle + a_1(|00...01\rangle + ... + |10...00\rangle)$ and

similarly for $|b\rangle$. Therefore, the problem of finding an entanglement witness for the produced state ρ is read as

$$\begin{aligned} \text{Find} \quad & \alpha\beta\gamma \\ \text{Subject to} \quad & \langle ab|\mathcal{W}_{\alpha\beta\gamma}|ab\rangle \geq 0 \\ \text{and} \quad & \text{tr}(\mathcal{W}_{\alpha\beta\gamma}\rho) < 0. \end{aligned} \tag{S13}$$

For $N = 8$, the number of parameters allows this problem to be solved numerically. The states we consider in the main text have the form

$$\rho = (1-q)\left[p|W_8\rangle\langle W_8| + (1-p)\frac{\mathcal{P}_1}{8}\right] + q\frac{\mathcal{P}_2}{28}. \tag{S14}$$

The condition for ρ being entangled then reads

$$(1-q)\left(\beta - \frac{7p+1}{8}\right) + \gamma q < 0, \tag{S15}$$

given that $\mathcal{W}_{\alpha\beta\gamma}$ is a witness. For small values of $1-p$ and q, it is possible to find such a witness. In particular, we numerically verify it for $p > 0.7$ and $q < 0.2$.

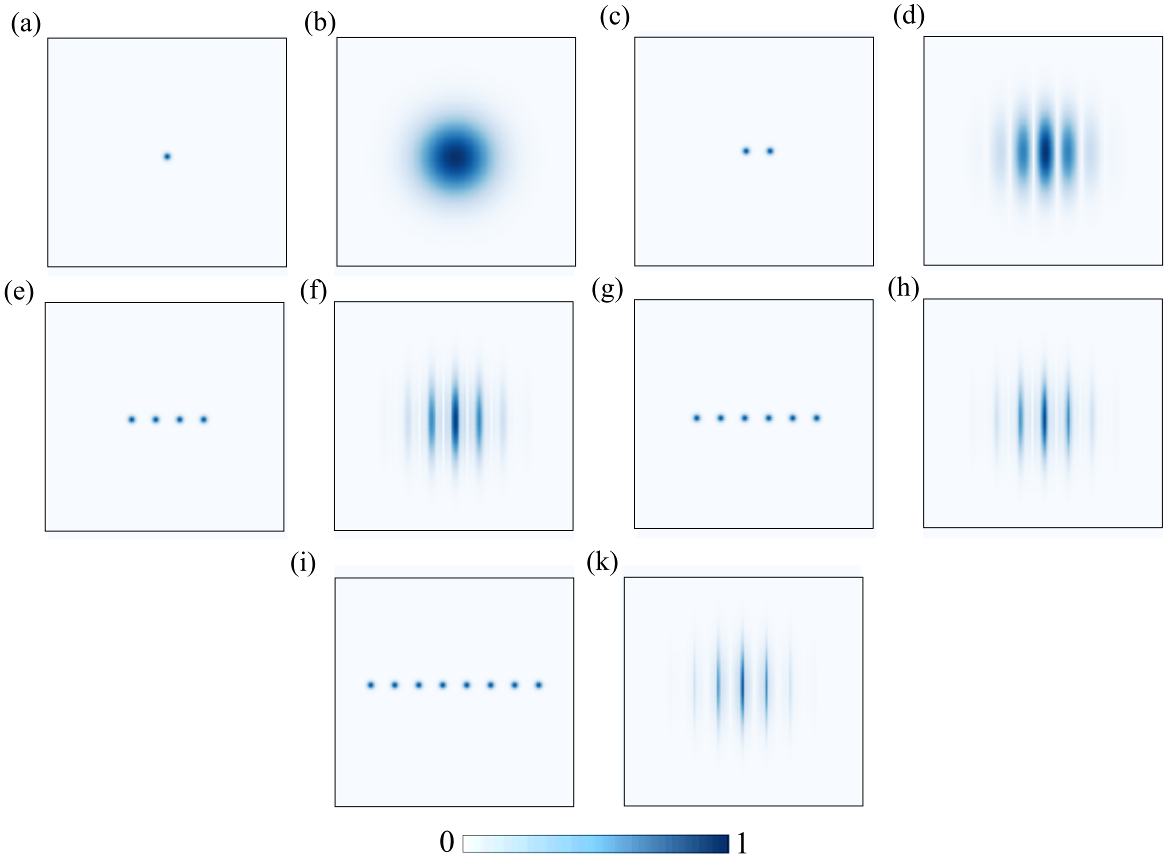

Figure S3: **W states in real and Fourier space**. (a), (c), (e), (g), (i) show real space images of W states of 1st, 2nd, 4th, 6th, and 8th order respectively. The corresponding Fourier transform images are shown in (b) ,(d) ,(f) ,(h) and (k). The simulated quantum states represent the ideal case of equal probability amplitudes in different channels, and selected relative phase difference of zero between them.

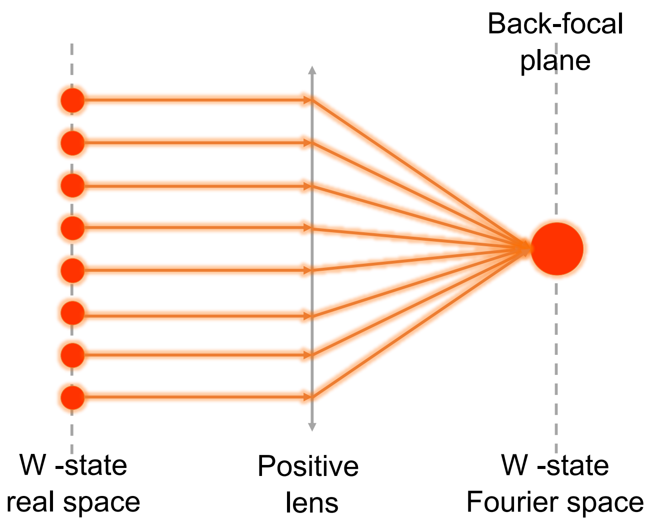

Figure S4: **Fourier transform using a positive lens**. Different modes in the W state are focused to the back-focal plane, with different path lengths corresponding to an overall phase factor of each mode.

References

[1] Chitambar, E., Leung, D., Mančinska, L., Ozols, M. & Winter, A. Everything you always wanted to know about locc (but were afraid to ask). *Communications in Mathematical Physics* **328**, 303–326 (2014).

[2] Nielsen, M. A. Conditions for a class of entanglement transformations. *Physical Review Letters* **83**, 436 (1999).

[3] de Vicente, J. I., Spee, C. & Kraus, B. Maximally entangled set of multipartite quantum states. *Physical review letters* **111**, 110502 (2013).

[4] Gühne, O. & Tóth, G. Entanglement detection. *Physics Reports* **474**, 1–75 (2009).

[5] Dür, W., Vidal, G. & Cirac, J. I. Three qubits can be entangled in two inequivalent ways. *Physical Review A* **62**, 062314 (2000).

[6] Horodecki, R., Horodecki, P., Horodecki, M. & Horodecki, K. Quantum entanglement. *Reviews of Modern Physics* **81**, 865 (2009).

[7] Cabello, A. Bell's theorem with and without inequalities for the three-qubit greenberger-horne-zeilinger and w states. *Physical Review A* **65**, 032108 (2002).

[8] Pan, J.-W., Bouwmeester, D., Daniell, M., Weinfurter, H. & Zeilinger, A. Experimental test of quantum nonlocality in three-photon greenberger–horne–zeilinger entanglement. *Nature* **403**, 515–519 (2000).

[9] Mermin, N. D. Extreme quantum entanglement in a superposition of macroscopically distinct states. *Physical Review Letters* **65**, 1838 (1990).

[10] Greenberger, D. M., Horne, M. A., Shimony, A. & Zeilinger, A. Bell's theorem without inequalities. *American Journal of Physics* **58**, 1131–1143 (1990).

[11] Wei, T.-C. & Goldbart, P. M. Geometric measure of entanglement and applications to bipartite and multipartite quantum states. *Physical Review A* **68**, 042307 (2003).

[12] Steinberg, J. & Gühne, O. Maximizing the geometric measure of entanglement. *arXiv preprint arXiv:2210.13475* (2022). [Online; accessed 24-Oct -2022].

[13] Dalacu, D. *et al.* Selective-area vapor-liquid-sold growth of tunable inasp quantum dots in nanowires. *Appl. Phys. Lett.* **98**, 251101 (2011).

[14] Dalacu, D. *et al.* Ultraclean emission from inasp quantum dots in defect-free wurtzite inp nanowires. *Nano letters* **12**, 5919–5923 (2012).

[15] Laferrière, P. *et al.* Unity yield of deterministically positioned quantum dot single photon sources. *Sci. Rep.* **12**, 6376 (2022).

[16] Häffner, H. *et al.* Scalable multiparticle entanglement of trapped ions. *Nature* **438**, 643–646 (2005).
